# The Association Between Psoriasis and Atherosclerotic Cardiovascular Disease: A Systematic Review and Meta-Analysis of Observational Studies

**DOI:** 10.7759/cureus.63379

**Published:** 2024-06-28

**Authors:** Pacelli C Osigwe, Chukwudike E Agomoh, Ifunanya S Osigwe, Florence K Akumiah

**Affiliations:** 1 Department of Cardiology, Bronglais General Hospital, Aberystwyth, GBR; 2 Department of Family Medicine, Hall Street Medical Centre, St. Helens, GBR; 3 Department of Medicine, Bronglais General Hospital, Aberystwyth, GBR; 4 Department of Medicine, Korle-Bu Teaching Hospital, Accra, GHA

**Keywords:** systematic review, psoriasis, observational studies, meta-analysis, cross-sectional studies, cohort studies, case-control studies, cardiovascular disease, atherosclerotic cardiovascular disease

## Abstract

Psoriasis is a chronic immune-mediated disease affecting the skin, nails, and/or joints. It is associated with systemic inflammation and may also be linked to an increased risk of atherosclerotic cardiovascular disease (ASCVD). The objectives of this study were to determine the overall risk of ASCVD in patients with psoriasis and to evaluate the risk according to ASCVD type and the severity of psoriasis. This was a systematic review and meta-analysis of observational studies reporting the association between psoriasis and one or more of the clinical types of ASCVD. We searched Medical Literature Analysis and Retrieval System Online (MEDLINE) via PubMed, Excerpta Medica Database (EMBASE), Scopus, Bielefeld Academic Search Engine (BASE), and Google Scholar for relevant studies in the English language from the beginning of their records to July 2023. Study selection and data extraction were conducted by four independent reviewers.

A total of 21 observational studies (three cross-sectional, one case-control, and 17 cohort) were included in this review, representing a total of 778,049 patients with psoriasis and 16,881,765 control subjects without psoriasis. The included studies had varying degrees of covariate adjustment, and thus, their findings may have been subject to residual confounding. All the meta-analyses used the adjusted effect sizes and were based on the random-effects model. However, the cohort studies were analysed separately from the non-cohort studies (the case-control and cross-sectional studies). There was a significant association between psoriasis and ASCVD (cohort studies: hazard ratio (HR), 1.21; 95% confidence interval (CI), 1.14 to 1.28; I^2^ = 63%; p < 0.001; non-cohort studies: odds ratio (OR), 1.60; 95% CI, 1.34 to 1.92; I^2^ = 31%; p = 0.23). Psoriasis was also significantly associated with myocardial infarction (cohort studies: HR, 1.20; 95% CI, 1.10 to 1.31; I^2^ = 60%; p < 0.001; non-cohort studies: OR, 1.57; 95% CI, 1.15 to 2.15; I^2^ = 74%; p = 0.05), coronary artery disease (cohort studies: HR, 1.20; 95% CI, 1.13 to 1.28; I^2^ = 67%; p < 0.001; non-cohort studies: OR, 1.60; 95% CI, 1.34 to 1.92; I^2^ = 31%; p = 0.23), aortic aneurysm (HR, 1.45; 95% CI, 1.04 to 2.02; I^2^ = 67%; p = 0.08) but not with ischaemic stroke (HR, 1.14; 95% CI, 0.96 to 1.36; I^2^ = 44%; p = 0.17). Pooled analysis in terms of the severity of psoriasis showed that both mild (cohort studies: HR, 1.17; 95% CI, 1.08 to 1.26; I^2^ = 74%; p < 0.001; non-cohort studies: OR, 1.54; 95% CI, 1.25 to 1.90; I^2^ = 0%; p = 0.50) and severe (cohort studies: HR, 1.43; 95% CI, 1.23 to 1.65; I^2^ = 65%; p < 0.001; non-cohort studies: OR, 1.65; 95% CI, 1.29 to 2.12; I^2^ = 25%; p = 0.26) psoriasis were significantly associated with ASCVD.

Psoriasis (including mild and severe disease) is associated with an increased risk of ASCVD, including coronary artery disease (CAD) and aortic aneurysm (AA). ASCVD risk assessment and prevention should be prioritised in all adult psoriasis patients. Future observational studies investigating the association between psoriasis and ASCVD should conduct a more comprehensive adjustment of covariates.

## Introduction and background

Psoriasis is a chronic immune-mediated inflammatory disorder of the skin, nails, and/or joints. This inflammatory condition has a global prevalence of 2-3% [1] and an estimated 60 million individuals are affected worldwide [2]. There is a significant geographical variation in the prevalence of psoriasis, with rates as high as 8-11% in the Scandinavian region [3,4] and as low as less than 1% in some Asian populations [5,6]. Generally, the incidence of psoriasis increases as one moves away from the equator [5]. Psoriasis is more common in adults, but children are also affected [5]. Males and females are equally affected [7-10], though multiple studies have reported a preponderance of males affected in East Asian populations [6,11-14].

Cardiovascular disease (CVD) is a group of disorders that affect the heart and/or the blood vessels. It accounts for more than four million deaths in Europe yearly [15] and about a third of all mortality worldwide [16]. Atherosclerotic cardiovascular disease (ASCVD) is the main subdivision of CVD and includes all CVDs related to atherosclerosis [15]. ASCVD is broadly classified into coronary artery disease (CAD) and peripheral artery disease (PAD) [15]. PAD is a kind of atherosclerotic disease that affects non-coronary sites such as the carotid arteries, vertebral arteries, retinal arteries, upper extremity arteries, aorta, renal arteries, mesenteric arteries, and lower extremity arteries [15].

Psoriasis may have a complex relationship with ASCVD. Atherosclerosis and psoriasis share the same T-helper 1 (Th1) and Th17 cell-mediated immune mechanisms, and their respective plaques have been shown to contain similar inflammatory cell infiltrates, cytokines, chemokines, adhesion molecules, and angiogenic/growth factors [17,18]. Psoriasis is associated with elevated inflammatory markers such as C-reactive protein [19,20], proving that chronic inflammation in this disorder is systemic and not just limited to the skin. Chronic immune-mediated inflammatory diseases may accelerate atherosclerosis through systemic inflammation, endothelial dysfunction, and the effects of anti-inflammatory medications used to treat them [21]. Furthermore, people with psoriasis have an increased prevalence of ASCVD risk factors, including hypertension, diabetes mellitus, dyslipidaemia, obesity, metabolic syndrome, smoking, excess alcohol consumption, and subclinical atherosclerosis [18,22-28]. However, the true nature of the relationship between psoriasis and ASCVD remains unresolved. The association between psoriasis and CVD has been evaluated in multiple observational studies. Although many of these studies [29-45] found significant associations, a good number of them [46-55] reported either conflicting or no associations.

A literature search showed that relatively recent systematic reviews and meta-analyses of observational studies [56-61] have evaluated the association between psoriasis and CVD. We decided to perform a new systematic review and meta-analysis of observational studies, limiting our study to the association between psoriasis and ASCVD. We also determined the association between psoriasis and individual ASCVD types, and the ASCVD association according to the severity of psoriasis. We intended our results to provide insight into the relevance of ASCVD preventive strategies (including lipid-lowering pharmacotherapy) in psoriasis patients.

## Review

Materials and methods

The exposure of interest in our review was psoriasis. Based on exposure status, two groups of individuals were involved in our study: individuals with psoriasis (exposed group) and individuals without psoriasis (unexposed or control group). The outcomes of interest were the different types of clinical ASCVD (CAD and PAD). We strictly adhered to the Preferred Reporting Items for Systematic Reviews and Meta-Analyses (PRISMA) guidelines [62].

Search Strategy

We conducted a thorough systematic literature search for relevant observational studies in five electronic databases, namely Medical Literature Analysis and Retrieval System Online (MEDLINE) through PubMed, Excerpta Medica Database (EMBASE), Scopus, Bielefeld Academic Search Engine (BASE), and Google Scholar. Their records were searched from the beginning until July 2023. The medical keywords/synonyms or medical subject headings (MeSH) terms allowed for search-building were slightly different from one database to another (see Appendix 1). Except for in BASE and Google Scholar, filters were added during the searches to limit the results as much as possible to human-only observational studies published in the English language. The reference lists of identified publications were manually searched to check for additional eligible publications.

Inclusion Criteria

To be included in our review, studies had to meet all of the following inclusion criteria: publication in the English language on or before July 2023; observational study design (cross-sectional, case-control, cohort, nested case-control, or nested case-cohort) of high-quality (score > 6) as assessed with the modified Newcastle-Ottawa tool for observational studies [63]; evaluation of one or more of the clinical types of ASCVD (CAD and/or PAD) in conjunction with psoriasis; and comparison of psoriasis patients with control groups in their analyses.

Exclusion Criteria

Observational studies meeting one or more of the following criteria were excluded from this review: exposed groups consisting only of patients with psoriatic arthritis, or patients with a particular psoriasis variant (for instance, plaque, scalp, nail, guttate, inverse, pustular, generalised pustular, palmoplantar, or erythrodermic psoriasis), or patients with a particular severity of psoriasis (mild, moderate, or severe); the inclusion of participants less than 18 years old (or where it was not clear that this age group was excluded from the study); the inclusion of participants from only one gender; the inclusion of animal participants; full-text unavailability; the absence of baseline data; the absence of an effect measure; and the absence of adjustment in the computed effect sizes.

Definition of Exposure and Outcomes

All exposure status (psoriasis) and ASCVD outcomes in this review were ascertained from reliable/reputable sources (medical databases, health insurance databases, national registries, and hospital records) mainly based on diagnostic codes such as the International Classification of Diseases (ICD), Oxford Medical Information System (OXMIS), and Read codes.

Classification of psoriasis into mild and severe disease was based mainly on the treatment modality, the body surface area (BSA) affected, or the Psoriasis Area and Severity Index (PASI) at skin examination. Patients with mild psoriasis required only topical medications for treatment or had ≤ 2% BSA involvement or a PASI score < 10. Patients with severe psoriasis had any one of the following characteristics: a history of hospitalisation for psoriasis, the use of systemic anti-psoriasis therapy (including phototherapy), the presence of psoriatic arthritis, > 10% BSA involvement, or a PASI score > 10. Some patients identified as having 'moderate' psoriasis due to 3-10% BSA involvement [64] were combined with the severe psoriasis group.

Clinical ASCVD outcomes included in our study were PAD (ischaemic stroke and aortic aneurysm) and CAD (including angina, myocardial infarction, and coronary revascularisation). Some studies used terms such as coronary heart disease (CHD) and ischaemic heart disease (IHD), which are synonyms for CAD.

Data Extraction and Study Quality Assessment

We assessed eligible studies for quality (risk of bias) using the modified Newcastle-Ottawa tool for observational studies [63]. Based on this tool, study quality was graded as high (if score > 6) or low (if score ≤ 6). Only high-quality studies were included in this review.

The following information was extracted from each included study: author name, year of publication, country of the study population, study design, the total number of study participants, the mean age of participants, gender distribution, mean follow-up time, number of psoriasis patients, number of control subjects, number of ASCVD outcomes in psoriasis and control groups, effect measure, adjusted effect size (including 95% confidence interval (CI)), and adjustment variables.

The processes of literature search, study selection, data extraction, and study quality assessment were done independently by four reviewers (PCO, CEA, ISO, and FKA), and all differences were resolved by consensus.

Statistical Analysis

Data was analysed using Comprehensive Meta-Analysis 3.0 (Biostat, Inc., Englewood, New Jersey, United States) and MetaXL (Microsoft Corporation, Redmond, Washington, United States). Effect measures were the hazard ratio (HR) and rate ratio (RR) for the cohort studies, and the odds ratio (OR) for the case-control and cross-sectional studies. RR was directly converted to HR in analyses because, in practical terms, both are similar [65]. Therefore, we analysed the cohort studies separately from the non-cohort studies, presenting pooled estimates as HR or OR, respectively.

The random-effects model was applied in all the meta-analyses. Heterogeneity among the included studies was assessed using the Q-statistic and I^2^ index, and the corresponding p-value. Heterogeneity was reported as low (I^2^ = 0% - 25%), moderate (I^2^ = 26% - 50%), or high (I^2^ > 50%). Sensitivity analysis was conducted when heterogeneity was high (I^2^ > 50%) to check the individual contributions of the studies included in the meta-analysis to the observed high heterogeneity. The results of the meta-analyses were presented using forest plots. The likelihood of publication bias was assessed visually using funnel plots and statistically using Egger’s regression test. All statistical tests were two-sided, with p < 0.05 considered statistically significant.

Ethical Considerations

This research was deemed as low risk and as such was reviewed by the Low-Risk Ethical Procedure Committee at the Faculty of Life Sciences and Education, University of South Wales, United Kingdom, and granted approval.

Results

Literature Search

**Figure 1:**
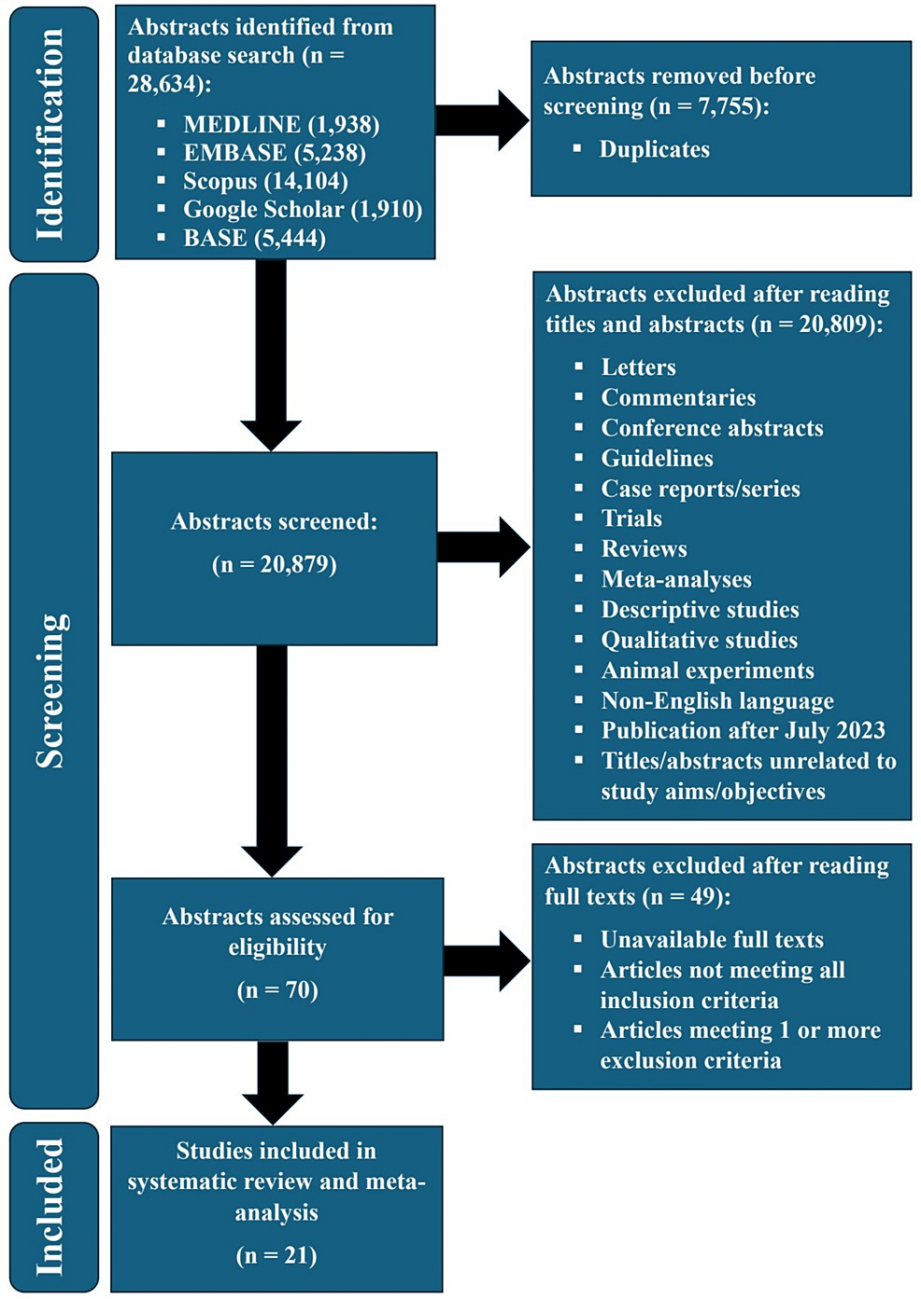
PRISMA flow diagram PRISMA: Preferred Reporting Items for Systematic Reviews and Meta-Analyses

From our search of five electronic databases (MEDLINE through PubMed, EMBASE, Scopus, BASE, and Google Scholar), we found 28,634 abstracts. The Rayyan software (Rayyan Systems, Inc., Cambridge, Massachusetts, United States) [66] was used to remove 7,755 duplicates from these search results. We proceeded to screen the remainder (20,879 abstracts) by reading them or their titles. This led to the exclusion of 20,809 abstracts due to their type/study design, date of publication, language, involvement of animals, or a lack of relationship with the aims/objectives of this systematic review. Next, the remaining 70 abstracts were taken for further review by retrieving their full texts. While reading the full text of each of these articles, we applied our inclusion and exclusion criteria to determine eligibility for inclusion in our study. Consequently, 21 observational studies (778,049 patients with psoriasis and 16,881,765 control subjects) were included in this systematic review and meta-analysis. Figure 1 shows the PRISMA flow diagram for this study.

Characteristics of the Included Studies

Of the 21 studies, three were cross-sectional studies, one was a case-control study, and the remaining 17 were cohort studies. Of the 17 cohort studies, one was a case-cohort study, seven were prospective cohort studies, and nine were retrospective cohort studies. In terms of the sampled population, five of the included studies were from North America (Canada and the United States), six were from East Asia (China, South Korea, and Taiwan), and the remaining 10 were from Europe (Denmark, the Netherlands, and the United Kingdom). Except for the case-control study [38] and one of the cross-sectional studies [67], all included studies were population-based.

The mean age of study participants at baseline ranged from 41 years [68] to 69 years [53]. Except for two [67,69], all studies from East Asia had greater proportions (up to 67%) of their study subjects as males, in keeping with the male predominance reported by multiple studies in this region [6,11-14]. By contrast, the participants in the European and American studies showed a roughly equal gender composition or a slight female predominance. The mean follow-up time for study participants in most of the cohort studies ranged from 3.3 years [70] to 13.8 years [71].

Fifteen of the studies (comprising three cross-sectional, one case-control, and 11 cohort) had baseline data that classified psoriasis groups into mild and severe subgroups based on the severity of the disease. Thirteen of these studies were population-based, and the proportion of psoriasis patients with severe disease ranged from 3% [29,43] to 27% [72,73]. The characteristics of the included studies are shown in Table 1.

**Table 1:** Characteristics of the included studies ^a^ mean follow-up for incident cardiovascular events (coronary heart disease, cerebral infarction, or heart failure); ^b^ psoriasis severity subgroups do not add up to the overall number because disease severity was not determined in all patients; ^c^ moderate psoriasis; ^d^ severe psoriasis; ^e^ other control groups were included in the computation AF, atrial fibrillation; AV, aortic valve; BMI, body mass index; BP, blood pressure; CABG, coronary artery bypass graft; CAD, coronary artery disease; CHF, congestive heart failure; CI, confidence interval; CKD, chronic kidney disease; Co, controls; COPD, chronic obstructive pulmonary disease; CVD, cardiovascular disease; DM, diabetes mellitus; HDL-C, high-density lipoprotein-cholesterol; HR, hazard ratio; IRR, incidence rate ratio; MI, myocardial infarction; N/A, not applicable; NR, not recorded; OR, odds ratio; PsO, psoriasis; PVD, peripheral vascular disease; RA, rheumatoid arthritis; RR, rate ratio; SBP, systolic blood pressure; SLE, systemic lupus erythematosus; TC, total cholesterol

Study reference	Country of study	Study design	Number of participants	Mean age, Years	Gender, Male%	Mean follow-up time, Years	Number of psoriasis cases			Number of controls	Number of outcomes in psoriasis cases			Number of outcomes in controls	Effect measure	Adjusted effect size (95% CI)			Adjustment variables
							Mild	Severe (Severe/ Overall %)	Overall		Mild	Severe	Overall			Mild	Severe	Overall	
Ischaemic stroke																			
Chiang et al. (2012) [39]	Taiwan	Retrospective cohort study	16,693	43.8	56.3	5			2,783	13,910			143	575	HR			1.27 (1.05–1.52)	Age, sex, and all comorbidities (hypertension, DM, dyslipidaemia, CKD, CAD, AF, valvular heart disease, SLE, and RA).
Dowlatshahi et al. (2013) [53]	The Netherlands	Prospective cohort study	8,190	68.6	42.2	10.9 ^a^	197	62 (23.9)	259	7,931			9	467	HR	0.62 (0.28–1.38)	0.93 (0.30–2.90)	0.69 (0.36–1.34)	Age, gender, current smoking, BMI, TC, HDL-C, SBP, antihypertensive medication, and DM.
Jung et al. (2019) [74]	South Korea	Retrospective cohort study	1,733,620	43.5	60.6	NR			5,788	1,727,832					HR	1.08 (0.93–1.27)	1.27 (0.93–1.74)		Age, sex, smoking status, alcohol consumption, exercise, BMI, dyslipidaemia, hypertension, and DM.
Coronary artery disease																			
Angina																			
Jung et al. (2019) [74]	South Korea	Retrospective cohort study	1,733,620	43.5	60.6	NR			5,788	1,727,832					HR	1.32 (1.17–1.50)	1.38 (1.06–1.79)		Age, sex, smoking status, alcohol consumption, exercise, BMI, dyslipidaemia, hypertension, and DM.
Myocardial infarction																			
Gelfand et al. (2006) [29]	United Kingdom	Prospective cohort study	687,971	45.9	47.1	5.4	127,139	3,837 (2.9)	130,976	556,995	2,319	112	2,431	11,194	HR	1.54 (1.24-1.91)	7.08 (3.06-16.36)		Hypertension, DM, cholesterol, age, gender, smoking, prior MI, and BMI.
Xiao et al. (2009) [67]	China	Cross-sectional study	4,613	44.5	49.6	N/A	1,619	1,473 (47.6)	3,092	1,521	97	118	215	45	Prevalence OR	1.72 (1.29–2.30)	2.01 (1.45–2.79)		Age, sex, the systemic treatment, obesity, DM, hypertension, hyperlipidemia, and smoking.
Wakkee et al. (2010) [50]	The Netherlands	Prospective cohort study	43,397	48.4	48.1	5.8	13,851	1,969 (12.4)	15,820	27,577			223	360	HR			0.94 (0.80 - 1.11)	Hypertension, DM, cholesterol, age, gender, and healthcare utilisation.
Ahlehoff et al. (2011) [36]	Denmark	Prospective cohort study	4,040,257	47.3	48.5	9.2	34,371	2,621 (7.1)	36,992	4,003,265					RR	1.22 (1.12–1.33)	1.45 (1.10–1.90)		Age, calendar year, concomitant medication, comorbidity (CHF, COPD, cardiac dysrhythmia, renal disease, cancer, rheumatological disease), socioeconomic data, and gender.
Lin et al. (2011) [69]	Taiwan	Retrospective cohort study	28,512	NR	49.7	5			4,752	23,760			22	48	HR			2.10 (1.27-3.43)	Hypertension, DM, hyperlipidaemia, and demographic risk factors (hospital clustering, monthly income, geographic region, and urbanisation level).
Levesque et al. (2013) [75]	Canada	Retrospective cohort study	62,842	52.8	46.6	4.3	26,262	5,159 (16.4)	31,421	31,421	468	110	578	796	HR	1.18 (1.05–1.33)	1.16 (0.94–1.42)	1.17 (1.04–1.31)	Age, DM, history of MI, hyperlipidaemia, hypertension, male sex, and use of corticosteroids.
Maradit-Kremers et al. (2013) [71]	United States	Retrospective, longitudinal cohort and nested case-cohort study	3,984	43.6	51	13.8			1,328	2,656			40	69	HR			1.18 (0.80 - 1.74)	Age, sex, and calendar year.
Yeung et al. (2013) [64]	United Kingdom	Cross-sectional study	99,385	46 (median)	47.4	10.7 (median)	4,523	3,122 (moderate PsO) and 1,081 (severe PsO) (12.4)	9,035 ^b^	90,350			95	693	Prevalence OR	1.39 (1.01–1.91)	1.39 (0.93–2.07) ^c^ 1.28 (0.68–2.44) ^d^	1.34 (1.07–1.69)	Age, sex, and years of follow-up.
Ogdie et al. (2015) [43]	United Kingdom	Prospective cohort study	219,997	48.4	47.4	5.3	134,095	4,329 (3.1)	138,424	81,573	1603	45	1643	838	HR	1.08 (0.98–1.18)	1.26 (0.92–1.72)		Age, sex, hypertension, DM, hyperlipidaemia, smoking status (never, past, current), and start year in the cohort.
Wu et al. (2015) [72]	United States	Retrospective cohort study	84,084	56.3	47.99	3.9	10,173	3,841 (27.4)	14,014	70,070	240	97	337	1,239	HR	1.31 (1.14-1.51)	1.28 (1.02-1.60)		Age, sex, DM, dyslipidaemia, hypertension, DM therapy, statin therapy, hypertension therapy, and beta-blocker therapy.
Egeberg et al. (2017) [76]	Denmark	Prospective cohort study	4,361,688	48.7	49.2	4.7	49,646	11,957 (19.4)	61,603	4,300,085	896	263	1,159	53,272	HR	1.03 (0.96–1.11)	1.21 (1.05–1.39)		Age, sex, socioeconomic status, previous CVD, DM, alcohol abuse, smoking, hypertension, cholesterol-lowering drug (statin) use, and healthcare consumption.
Leisner et al. (2018) [77] (early era cohort)	Denmark	Prospective cohort study	48,093	NR	47	5			4,302	43,791					HR			1.40 (1.09-1.80)	Birth year, sex, education, and use of CVD-related drugs.
Leisner et al. (2018) [77] (late era cohort)	Denmark	Prospective cohort study	50,953	NR	47	5			4,577	46,376					HR			1.39 (1.10-1.75)	Birth year, sex, education, and use of CVD-related drugs.
Jung et al. (2019) [74]	South Korea	Retrospective cohort study	1,733,620	43.5	60.6	NR			5,788	1,727,832					HR	0.96 (0.72 –1.26)	2.24 (1.51–3.32)		Age, sex, smoking status, alcohol consumption, exercise, BMI, dyslipidaemia, hypertension, and DM.
Coronary artery disease																			
Armstrong et al. (2012) [38]	United States	Case-control study	9,469	61	59.3	N/A	153	51 (25)	204	9,265			172	7,020	OR			1.84 (1.19–2.83)	Age, ethnicity, gender, BMI, history of DM, cerebrovascular disease, PVD, hypertension, smoking status, previous MI, hypercholesterolaemia, previous CABG, and family history of CAD.
Coronary heart disease																			
Dowlatshahi et al. (2013) [53]	The Netherlands	Prospective cohort study	8,190	68.6	42.2	10.9 ^a^	197	62 (23.9)	259	7,931			14	812	HR	0.85 (0.49–1.47)	0.21 (0.03–1.50)	0.70 (0.41–1.19)	Age, gender, current smoking, BMI, TC, HDL-C, SBP, antihypertensive medication, and DM.
Dregan et al. (2014) [78]	United Kingdom	Retrospective cohort study	up to 272,640	46.8 ^e^	43.9 ^e^	NR	85,232	5,648 (6.2)	90,880	up to 181,760	1,406	134	1,540	2,557	HR	1.03 (0.97–1.11)	1.29 (1.01–1.64)		Age, age squared, sex, BP, cholesterol, BMI, smoking, alcohol, serum creatinine levels, glucocorticoids, antihypertensive medications, and statins.
Feldman et al. (2018) [70]	United States	Retrospective cohort study	229,648	53	46.2	3.3			114,824	114,824					HR			1.23 (1.19-1.27)	Baseline Deyo-Charlson Comorbidity Index score, index year, and insurance plan type.
Ischaemic heart disease																			
Wakkee et al. (2010) [50]	The Netherlands	Prospective cohort study	43,397	48.4	48.1	5.7	13,851	1,969 (12.4)	15,820	27,577			583	846	HR			1.05 (0.95 - 1.17)	Hypertension, DM, cholesterol, age, gender, and healthcare utilisation.
Yang et al. (2011) [35]	Taiwan	Cross-sectional study	6,740	48·6	67.4	N/A	1,384	301 (17.9)	1,685	5,055			27	52	OR	1·04 (0·32–3·36)	1·61 (0·98–2·63)	1·51 (1·02–2·43)	Monthly income, geographical region, and level of urbanisation of the patient’s community.
Jung et al. (2019) [74]	South Korea	Retrospective cohort study	1,733,620	43.5	60.6	NR			5,788	1,727,832					HR	1.25 (1.11 –1.41)	1.52 (1.21–1.92)		Age, sex, smoking status, alcohol consumption, exercise, BMI, dyslipidaemia, hypertension, and DM.
Coronary revascularisation																			
Ahlehoff et al. (2011) [36]	Denmark	Prospective cohort study	4,040,257	47.3	48.5	9.2	34,371	2,621 (7.1)	36,992	4,003,265					RR	1.37 (1.26–1.49)	1.77 (1.35–2.32)		Age, calendar year, concomitant medication, comorbidity (CHF, COPD, cardiac dysrhythmia, renal disease, cancer, rheumatological disease), socioeconomic data, and gender.
Aortic aneurysm																			
Chiu et al. (2016) [73]	Taiwan	Retrospective cohort study	171,505	42.8	57.2	6.5	24,963	9,338 (27.2)	34,301	137,204	36	15	51	94	HR	1.73 (1.12‐2.65)	2.15 (1.03‐4.51)	1.80 (1.25‐2.61)	Cardiovascular conditions, comorbidities (hypertension, hyperlipidaemia, DM, CKD, atherosclerosis, stroke, bicuspid AV, stenosis of carotid or peripheral artery, RA, obesity, alcohol dependence, and tobacco use disorder), and medication use during the preceding year.
Khalid et al. (2016) [68]	Denmark	Retrospective cohort study	5,475,533	41.0	49.3	11.7	59,423	11,566 (16.3)	70,989	5,404,544	240	50	290	23,696	IRR	1.20 (1.03‐1.39)	1.67 (1.21‐2.32)	1.27 (1.11‐1.46)	Age, sex, calendar year, comorbidity, concomitant medications (AF, DM, hypertension, vascular disease, and thromboembolism), socioeconomic status, and smoking history.

Quality Assessment

We performed a quality assessment of the 21 included studies using the modified Newcastle-Ottawa tool for observational studies [63]. This tool has separate scales for the three types of observational studies (cross-sectional, case-control, and cohort studies). All 21 studies had quality scores of at least seven, and were, thus, deemed high quality.

All three cross-sectional studies had a quality score of nine out of a maximum score of 10, with a mean score of nine (standard deviation ± 0). The quality score for the case-control study was eight out of a maximum score of nine. Out of a maximum score of nine, the 17 cohort studies had quality scores ranging from seven to nine, with a mean score of eight (standard deviation ± 0.35). Tables 2-4 summarise the quality assessment of the 21 studies.

**Table 2:** Quality assessment of the included cross-sectional studies (-) no mark; (+) one mark; (++) two marks.

Study reference	Representativeness of the sample (maximum score = 1)	Sample size (maximum score = 1)	Non-respondents (maximum score = 1)	Ascertainment of exposure (maximum score = 2)	The subjects in different outcome groups are comparable, based on the study design or analysis. Confounding factors are controlled (maximum score = 2)	Assessment of the outcome (maximum score = 2)	Statistical test (maximum score = 1)	Quality score (maximum score = 10)
Xiao et al., 2009 [67]	+	-	+	++	++	++	+	9
Yang et al., 2011 [35]	+	-	+	++	++	++	+	9
Yeung et al., 2013 [64]	+	+	-	++	++	++	+	9

**Table 3:** Quality assessment of the included case-control study (-) no mark; (+) one mark; (++) two marks.

Study reference	Is the case definition adequate? (maximum score = 1)	Representativeness of the cases (maximum score = 1)	Selection of controls (maximum score = 1)	Definition of controls (maximum score = 1)	Comparability of cases and controls on the basis of the design or analysis (maximum score = 2)	Ascertainment of exposure (maximum score = 1)	Same method of ascertainment for cases and controls (maximum score = 1)	Non-response rate (maximum score = 1)	Quality score (maximum score = 9)
Armstrong et al., 2012 [38]	+	+	+	+	++	+	+	-	8

**Table 4:** Quality assessment of the included cohort studies (-) no mark; (+) one mark; (++) two marks.

Study reference	Representativeness of the exposed cohort (maximum score = 1)	Selection of the non-exposed cohort (maximum score = 1)	Ascertainment of exposure (maximum score = 1)	Demonstration that outcome of interest was not present at the start of the study (maximum score = 1)	Comparability of cohorts on the basis of the design or analysis (maximum score = 2)	Assessment of outcome (maximum score = 1)	Was follow-up long enough for outcomes to occur? (maximum score = 1)	Adequacy of follow-up of cohorts (maximum score = 1)	Quality score (maximum score = 9)
Gelfand et al. (2006) [29]	+	+	+	+	++	+	+	-	8
Wakkee et al. (2010) [50]	_+_	+	+	+	++	+	+	-	8
Ahlehoff et al. (2011) [36]	+	+	+	+	++	+	+	-	8
Lin et al. (2011) [69]	+	+	+	+	++	+	+	-	8
Chiang et al. (2012) [39]	+	+	+	+	++	+	+	-	8
Dowlatshahi et al. (2013) [53]	+	+	+	+	++	+	+	-	8
Levesque et al. (2013) [75]	+	+	+	+	++	+	+	-	8
Maradit-Kremers et al. (2013) [71]	+	+	+	+	+	+	+	-	7
Dregan et al. (2014) [78]	+	+	+	+	++	+	+	+	9
Ogdie et al. (2015) [43]	+	+	+	+	++	+	+	-	8
Wu et al. (2015) [72]	+	+	+	+	++	+	+	-	8
Chiu et al. (2016) [73]	+	+	+	+	++	+	+	-	8
Khalid et al. (2016) [68]	+	+	+	+	++	+	+	-	8
Egeberg et al. (2017) [76]	+	+	+	+	++	+	+	-	8
Feldman et al. (2018) [70]	+	+	+	+	++	+	+	-	8
Leisner et al. (2018) [77]	+	+	+	+	+	+	+	+	8
Jung et al. (2019) [74]	+	+	+	-	++	+	+	+	8

Publication Bias

**Figure 2:**
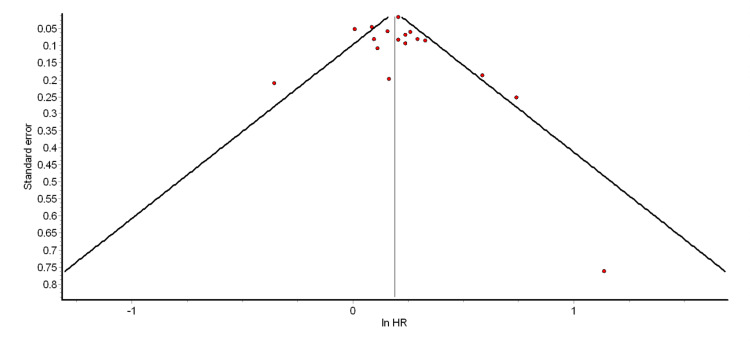
Funnel plot for the pooled analysis of overall ASCVD (cohort studies) ASCVD: atherosclerotic cardiovascular disease; HR: hazard ratio

**Figure 3:**
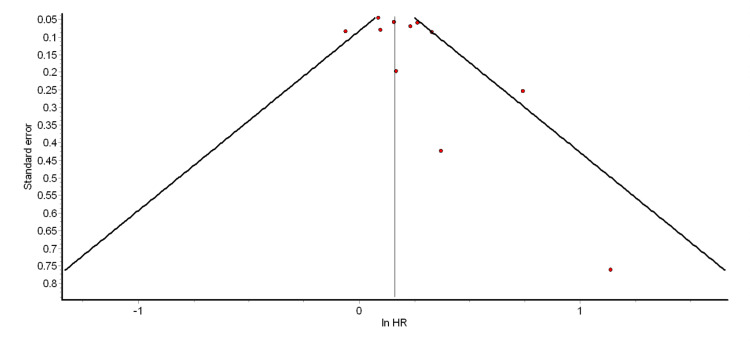
Funnel plot for the pooled analysis of MI (cohort studies) MI: myocardial infarction; HR: hazard ratio

**Figure 4:**
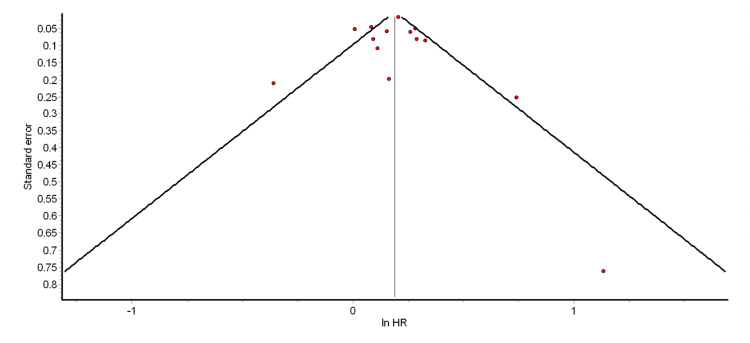
Funnel plot for pooled analysis of CAD (cohort studies) CAD: coronary artery disease; HR: hazard ratio

**Figure 5:**
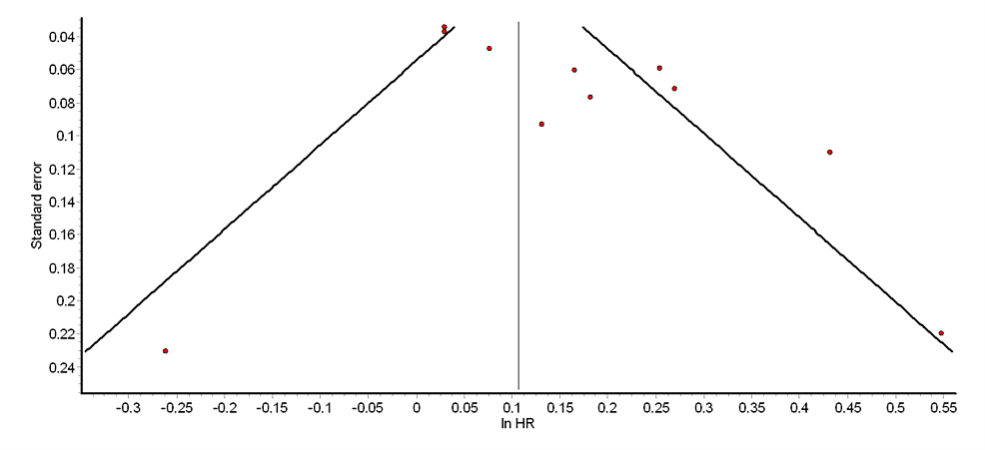
Funnel plot for the pooled analysis of mild psoriasis and ASCVD (cohort studies) ASCVD: atherosclerotic cardiovascular disease; HR: hazard ratio

**Figure 6:**
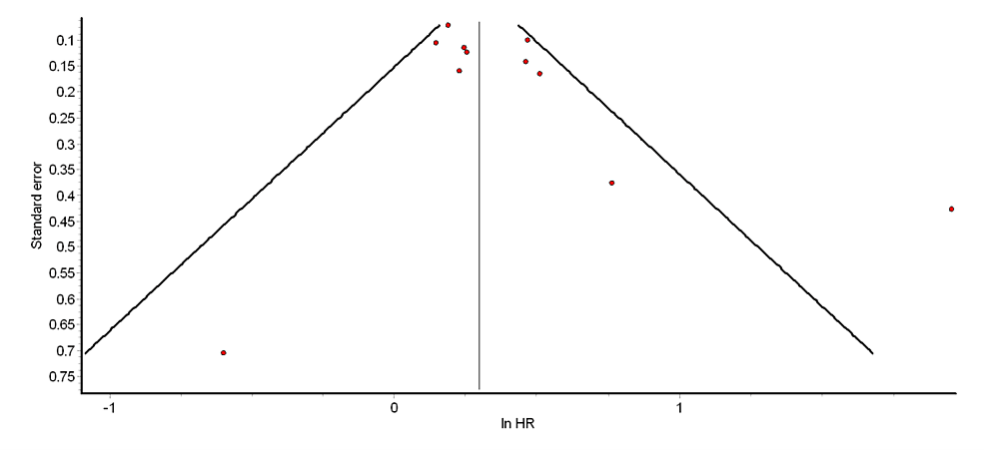
Funnel plot for the pooled analysis of severe psoriasis and ASCVD (cohort studies) ASCVD: atherosclerotic cardiovascular disease; HR: hazard ratio

The funnel plot and Egger’s test were used to test for the presence of significant publication bias. The funnel plots for the different pooled analyses we performed (Figures 2-6 and Appendix 2) were asymmetrical, suggesting the presence of publication bias. The power of Egger’s test to detect publication bias is greater when at least 10 studies are included in a meta-analysis [79]. Thus, we ran Egger’s tests for the pooled analyses of overall ASCVD (cohort studies) (p = 0.74), myocardial infarction (cohort studies) (p = 0.35), CAD (cohort studies) (p = 0.98), mild psoriasis and ASCVD (cohort studies) (p = 0.15), and severe psoriasis and ASCVD (cohort studies) (p = 0.14). The results showed that the asymmetries observed in the respective funnel plots (Figures 2-6) were not due to publication bias.

Pooled Analyses

Pooled analyses were done for overall ASCVD, ischaemic stroke, myocardial infarction (MI), CAD, and aortic aneurysm (AA). Pooled analysis was also done in terms of the severity of psoriasis.

Pooled analysis of overall ASCVD: A total of 21 studies investigated the risk of one or more ASCVD outcomes, including ischaemic stroke, angina, MI, CAD, CHD, IHD, coronary revascularisation, and AA. The 21 studies included a total of 778,049 patients with psoriasis and 16,881,765 control subjects without psoriasis.

**Figure 7:**
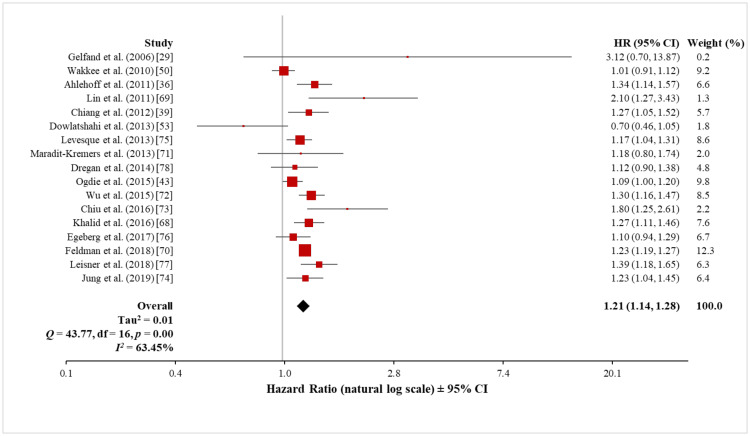
Forest plot of the association between psoriasis and ASCVD (cohort studies) ASCVD: atherosclerotic cardiovascular disease

Meta-analysis of the 17 cohort studies (764,033 patients with psoriasis and 16,775,574 control subjects) using the random-effects model showed a significantly increased overall risk of ASCVD in patients with psoriasis (HR, 1.21; 95% CI, 1.14 to 1.28) (Figure 7). The heterogeneity test showed a significant difference among these 17 studies (Q = 43.77 with 16 df, p < 0.001; I^2^ = 63%). Sensitivity analysis, with the exclusion of the data set of one study at a time, did not significantly affect the observed heterogeneity (see Appendix 3). Therefore, although the meta-analysis showed a moderately increased overall risk of ASCVD, there is a need for a careful interpretation of this result due to the high heterogeneity.

**Figure 8:**
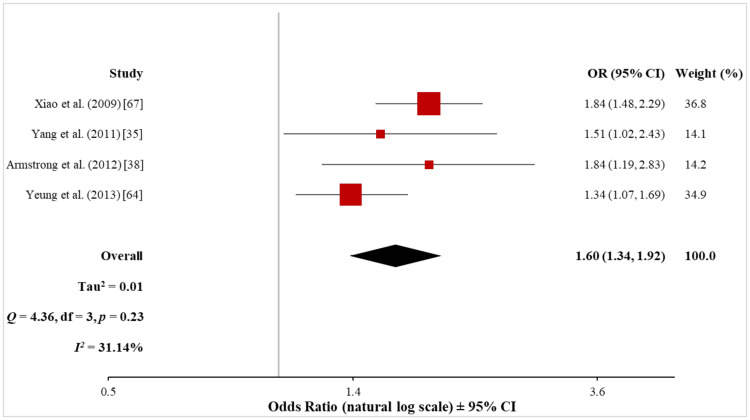
Forest plot of the association between psoriasis and ASCVD (non-cohort studies) ASCVD: atherosclerotic cardiovascular disease

Meta-analysis of the four non-cohort studies (14,016 patients with psoriasis and 106,191 control subjects) using the random-effects model showed a significantly increased overall risk of ASCVD in patients with psoriasis (OR, 1.60; 95% CI, 1.34 to 1.92) (Figure 8). There was moderate heterogeneity (Q = 4.36 with 3 df, p = 0.23; I^2^ = 31%), which implies no significant difference among these four studies.

**Figure 9:**
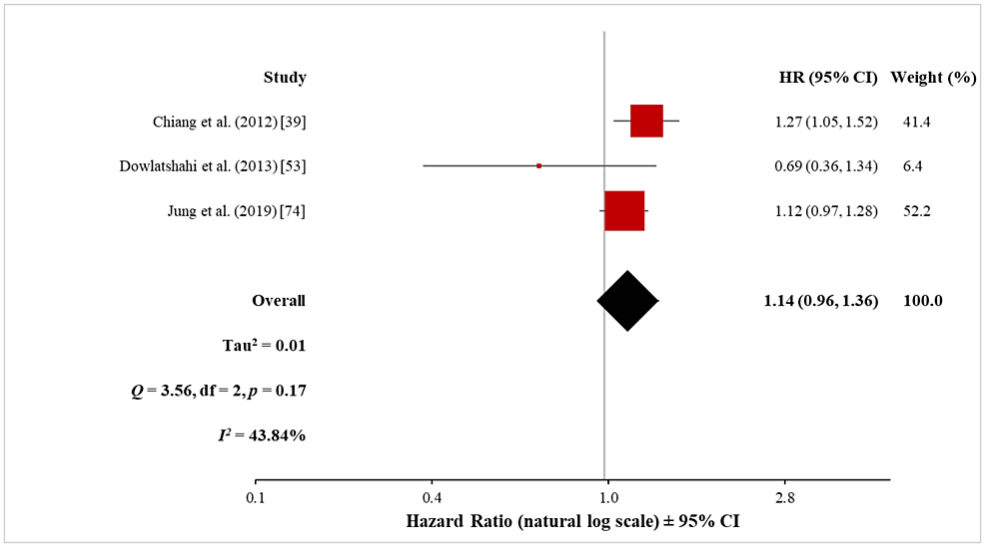
Forest plot of the association between psoriasis and ischaemic stroke

Pooled analysis of ischaemic stroke: Three cohort studies investigated the risk of ischaemic stroke in patients with psoriasis, including 8,830 psoriasis patients and 1,749,673 control subjects without psoriasis. The meta-analysis with the random-effects model did not show a significantly increased risk of ischaemic stroke in patients with psoriasis (HR, 1.14; 95% CI, 0.96 to 1.36) (Figure 9). The moderate heterogeneity (Q = 3.56 with 2 df, p = 0.17; I^2^ = 44%) showed there was no significant difference among these three studies.

Pooled analysis of MI: A total of 13 studies investigated the risk of MI in patients with psoriasis, including 462,124 psoriasis patients and 11,007,272 control subjects without psoriasis. Eleven of these studies were cohort studies while two were cross-sectional studies.

**Figure 10:**
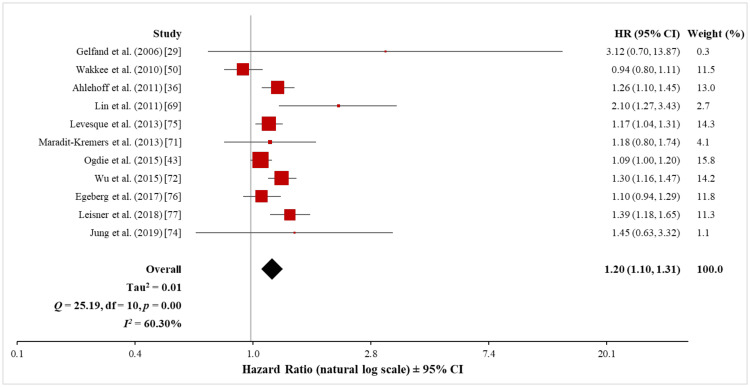
Forest plot of the association between psoriasis and MI (cohort studies) MI: myocardial infarction

The meta-analysis of the 11 cohort studies (449,997 patients with psoriasis and 10,915,401 control subjects) using the random-effects model showed a significantly increased risk of MI in patients with psoriasis (HR, 1.20; 95% CI, 1.10 to 1.31) (Figure 10). Heterogeneity testing revealed a significant difference among these 11 studies (Q = 25.19 with 10 df, p < 0.001; I^2^ = 60%). Although the exclusion of the data set from Wakkee et al. [50] significantly reduced the observed heterogeneity to a moderate level (I^2^ = 48%) without affecting the significant association between psoriasis and MI (HR, 1.23; 95% CI, 1.13 to 1.33), the Q-statistic still suggested statistically significant heterogeneity (Q = 17.46 with 9 df, p = 0.04) (see Appendix 3).

**Figure 11:**
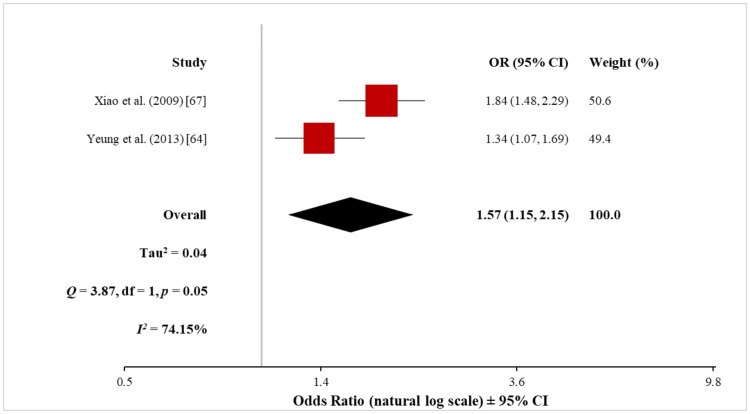
Forest plot of the association between psoriasis and MI (non-cohort studies) MI: myocardial infarction

The meta-analysis of the two non-cohort studies (12,127 patients with psoriasis and 91,871 control subjects) using the random-effects model showed a significantly increased risk of MI in patients with psoriasis (OR, 1.57; 95% CI, 1.15 to 2.15) (Figure 11). However, heterogeneity testing revealed a significant difference between these two studies (Q = 3.87 with 1 df, p = 0.05; I^2^ = 74%), necessitating a cautious interpretation of the result of the meta-analysis.

Pooled analysis of CAD: A total of 18 studies investigated the risk of CAD outcomes (angina, MI, CAD, CHD, IHD, and coronary revascularisation) in patients with psoriasis. They included a total of 669,976 psoriasis patients and 11,326,107 control subjects without psoriasis. Fourteen of these studies were cohort studies, one was a case-control study, and the remaining three were cross-sectional studies.

**Figure 12:**
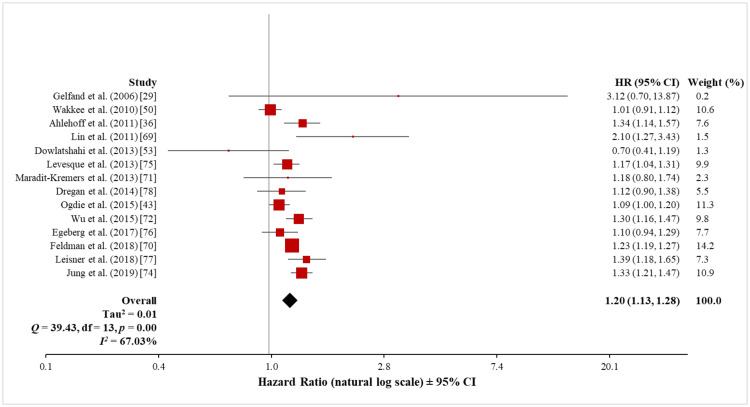
Forest plot of the association between psoriasis and CAD (cohort studies) CAD: coronary artery disease

The meta-analysis of the 14 cohort studies (655,960 patients with psoriasis and 11,219,916 control subjects) using the random-effects model showed a significantly increased risk of CAD in patients with psoriasis (HR, 1.20; 95% CI, 1.13 to 1.28) (Figure 12). The heterogeneity test showed a significant difference among these 14 studies (Q = 39.43 with 13 df, p < 0.001; I^2^ = 67%). Sensitivity analysis, with the exclusion of the data set of one study at a time, did not significantly affect the observed heterogeneity (see Appendix 3). Because of this high heterogeneity, the findings of the meta-analysis should be interpreted carefully.

**Figure 13:**
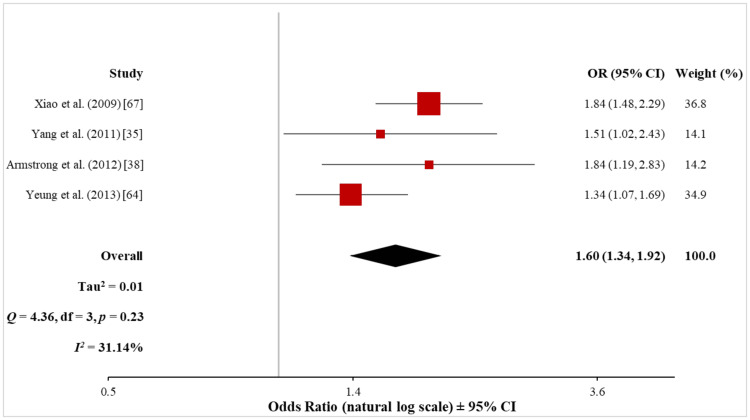
Forest plot of the association between psoriasis and CAD (non-cohort studies) CAD: coronary artery disease

The meta-analysis of the four non-cohort studies (14,016 patients with psoriasis and 106,191 control subjects) using the random-effects model showed a significantly increased risk of CAD in patients with psoriasis (OR, 1.60; 95% CI, 1.34 to 1.92) (Figure 13). Heterogeneity was only moderate (Q = 4.36 with 3 df, p = 0.23; I^2^ = 31%), which showed that there was no significant difference among these four studies.

**Figure 14:**
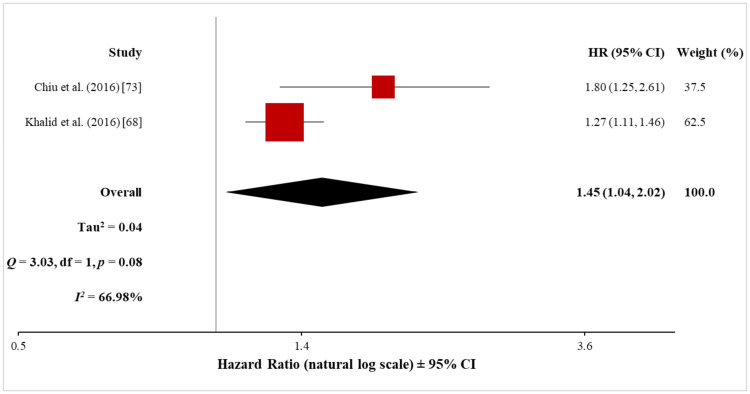
Forest plot of the association between psoriasis and AA AA: aortic aneurysm

Pooled analysis of AA: Two cohort studies investigated the risk of AA in patients with psoriasis, including 105,290 psoriasis patients and 5,541,748 control subjects without psoriasis. Meta-analysis with the random-effects model showed a significantly increased risk of AA in patients with psoriasis (HR, 1.45; 95% CI, 1.04 to 2.02) (Figure 14). The heterogeneity test showed that there was a significant difference between these two studies (Q = 3.03 with 1 df, p = 0.08; I^2^ = 67%). Again, this high heterogeneity makes cautious interpretation of the meta-analysis result necessary.

Pooled analysis in terms of severity of psoriasis: A total of 14 studies determined the risk of ASCVD outcome according to the severity of psoriasis (mild and severe psoriasis). These studies included a total of 629,459 patients with psoriasis and 16,599,606 control subjects without psoriasis. Eleven of these studies were cohort studies, while the rest were cross-sectional studies.

**Figure 15:**
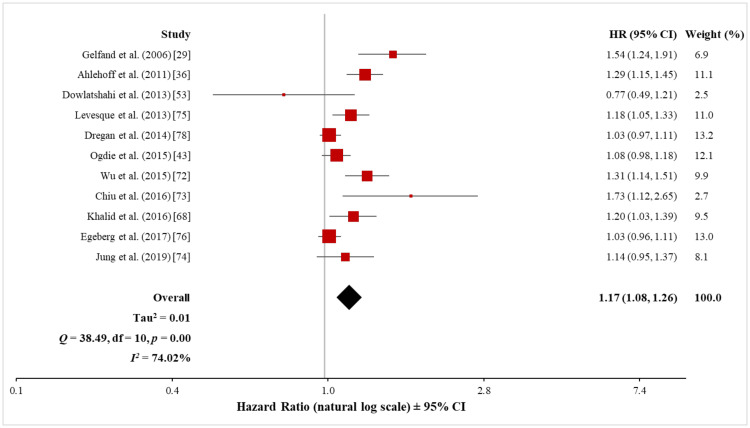
Forest plot of the association between mild psoriasis and ASCVD (cohort studies) ASCVD: atherosclerotic cardiovascular disease

**Figure 16:**
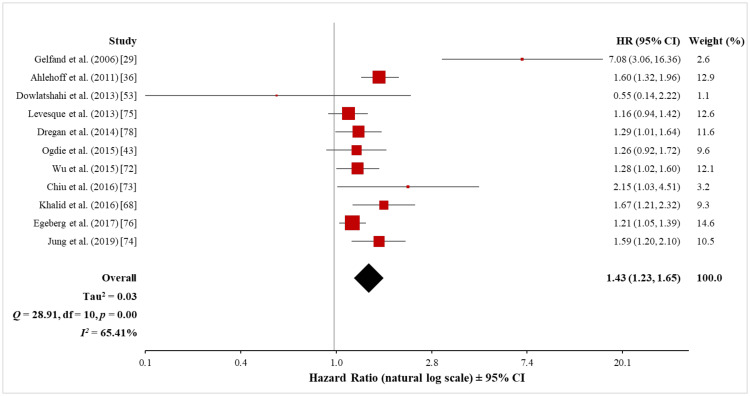
Forest plot of the association between severe psoriasis and ASCVD (cohort studies) ASCVD: atherosclerotic cardiovascular disease

The meta-analysis of the 11 cohort studies (615,647 psoriasis patients and 16,502,680 control subjects) using the random-effects model showed a significantly increased risk of ASCVD in both mild (HR, 1.17; 95% CI, 1.08 to 1.26) and severe (HR, 1.43; 95% CI, 1.23 to 1.65) psoriasis patients (Figures 15, 16). However, when tested for heterogeneity, there was a significant difference among the studies included in the meta-analysis both for mild (Q = 38.49 with 10 df, p < 0.001; I^2^ = 74%) and severe (Q = 28.91 with 10 df, p < 0.001; I^2^ = 65%) psoriasis. Sensitivity analysis, with the exclusion of one study at a time, did not significantly affect the observed heterogeneity of the pooled analysis in terms of mild psoriasis (see Appendix 3). However, in the sensitivity analysis of the pooled analysis in terms of severe psoriasis, the exclusion of the data set from Gelfand et al. [29] significantly reduced the observed heterogeneity (Q = 13.72 with 9 df, p = 0.13; I^2^ = 34%) while maintaining a significant association between severe psoriasis and ASCVD (HR, 1.35; 95% CI, 1.22 to 1.50) (see Appendix 3). Thus, a definite conclusion can only be drawn from the pooled analysis in terms of severe psoriasis.

**Figure 17:**
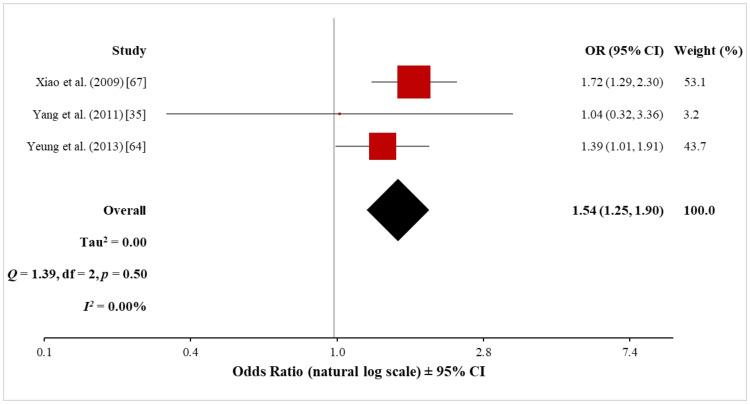
Forest plot of the association between mild psoriasis and ASCVD (non-cohort studies) ASCVD: atherosclerotic cardiovascular disease

**Figure 18:**
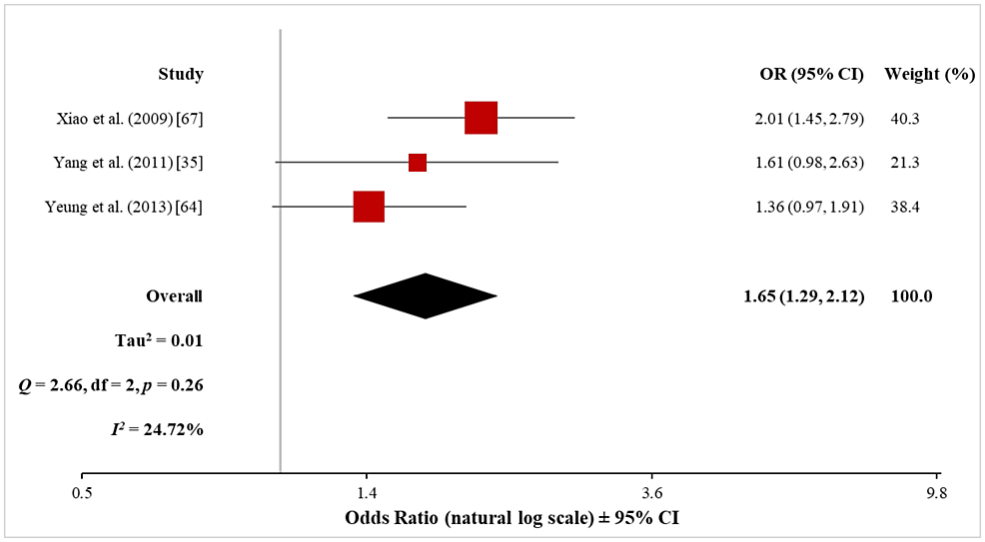
Forest plot of the association between severe psoriasis and ASCVD (non-cohort studies) ASCVD: atherosclerotic cardiovascular disease

The meta-analysis of the three non-cohort studies (13,812 psoriasis patients and 96,926 control subjects) using the random-effects model showed a significantly increased risk of ASCVD in both mild (OR, 1.54; 95% CI, 1.25 to 1.90) and severe (OR, 1.65; 95% CI, 1.29 to 2.12) psoriasis patients (Figures 17, 18). Heterogeneity was low both for mild (Q = 1.39 with 2 df, p = 0.50; I^2^ = 0%) and severe (Q = 2.66 with 2 df, p = 0.26; I^2^ = 25%) psoriasis, which suggested that there was no significant difference among the studies included in the respective meta-analyses.

Discussion

The results of our meta-analyses showed that psoriasis was associated with a significantly increased risk of ASCVD. There were significant associations with MI, CAD, and AA, but not with ischaemic stroke. Meta-analyses also showed that both mild and severe psoriasis were associated with an increased risk of ASCVD.

Liu et al. [61] conducted a systematic review and meta-analysis of 31 cohort studies; 15 of which met the inclusion criteria of our meta-analysis. In their analysis, patients with psoriasis had a significantly increased risk of developing adverse cardiovascular outcomes, including stroke (RR, 1.19; 95% CI, 1.11 to 1.27), MI (RR, 1.17; 95% CI, 1.11 to 1.24), IHD (RR, 1.17; 95% CI 1.02 to 1.34), thromboembolism (RR, 1.36; 95% CI, 1.20 to 1.55), arrhythmia (RR, 1.35; 95% CI, 1.30 to 1.40), and cardiovascular death (RR, 1.46; 95% CI, 1.26 to 1.69). Similar positive associations between psoriasis and CVD outcomes were also reported in systematic reviews and meta-analyses by Xu and Zhang [56], Armstrong et al. [57], Horreau et al. [58], Miller et al. [59], Samarasekera et al. [60], and Yu et al. [80]. Like in our study, high heterogeneity was observed in the analyses of most of these reviews.

The presence of an association between psoriasis and atherosclerosis has a biologically plausible explanation. Psoriasis is no longer considered a local inflammatory condition limited to the skin because the presence of systemic inflammation has been demonstrated [19,20]. Systemic inflammation and endothelial dysfunction in chronic immune-mediated inflammatory diseases are known to accelerate atherosclerosis [21]. As earlier stated, both psoriasis and atherosclerosis have the same underlying Th1 and Th17 cell-mediated immune mechanisms, and their chronic inflammatory plaques have similar inflammatory cell and cytokine constituents [17,18]. Therefore, the chronic systemic inflammation in psoriasis may be an important driver of atherosclerosis, which partly explains the positive association we found between psoriasis and ASCVD. Adiponectin, a cytokine secreted by adipose tissue, has anti-inflammatory actions that inhibit the initiation and progression of atherosclerosis [81]. To further support the 'inflammation theory', a cross-sectional observational study [82] found that individuals with psoriasis had significantly lower serum adiponectin levels compared with those without psoriasis, regardless of the presence of metabolic syndrome.

ASCVD risk factors such as smoking, hypertension, diabetes mellitus, dyslipidaemia, and obesity are highly prevalent in patients with psoriasis [18]. Depression and other mental illnesses (non-traditional ASCVD risk factors) are also associated with psoriasis [83,84]. All the included studies in our review performed incomplete adjustment for ASCVD risk factors (and other covariates), although some conducted more extensive adjustment than others (Table 1). Hence, residual confounding, which cannot be ruled out in these studies, may be another mechanism that partly explains the results of our meta-analyses.

Anti-psoriasis treatment, an important covariate, was adjusted for by only a minority of the included studies (Table 1). Treatment options for psoriasis include topical medications, phototherapy, and systemic medications. Systemic medications used by psoriasis patients in the included studies were methotrexate, azathioprine, hydroxyurea, mycophenolate, ciclosporin, retinoids (etretinate or acitretin), and biological drugs such as alefacept, efalizumab, adalimumab, etanercept, infliximab, and ustekinumab. Studies [85,86] have suggested that methotrexate and the tumour necrosis factor (TNF)-alpha inhibitors may reduce the risk of CVD, including MI, in patients with psoriasis. Treating psoriasis with TNF-alpha inhibitors may also reduce the incidence of diabetes mellitus [87], while other systemic medications such as acitretin and ciclosporin may lead to hyperlipidaemia [60]. However, the effects on ASCVD of many of the utilised systemic medications in our included studies remain undetermined but may be direct (independent), indirect, or no effect. Given that most studies did not adjust for anti-psoriasis treatment, it is likely that systemic medications impacted the findings of our review, although the exact nature of this impact remains unknown. Further research with studies of high levels of evidence (such as randomised controlled trials (RCTs)) is necessary to resolve the uncertainty of the effects of systemic anti-psoriasis medications on ASCVD.

Except for ischaemic stroke, psoriasis had significant associations with all our clinical ASCVD outcomes. Some of these outcomes (ischaemic stroke and AA) represent heterogeneous disorders that also have non-atherosclerotic causes. For instance, nearly half of all ischaemic strokes are related to cardioembolism or have an undetermined cause [88]. This heterogeneity may partly account for the result of our pooled analysis of ischaemic stroke which did not show a significant association.

The findings of our study have important implications for clinical practice. As the meta-analyses suggest an independent positive association between psoriasis and ASCVD, it may be prudent for clinicians (including general practitioners, dermatologists, and rheumatologists) who provide care for adult psoriasis patients to regard psoriasis as a risk modifier that upgrades an individual's CVD risk category. Re-categorisation affects the recommended low-density lipoprotein cholesterol (LDL-C) target for CVD prevention. It could also influence clinical decisions regarding the need for lipid-lowering therapy, in addition to lifestyle modification, for the prevention of CVD. Given that there is also an association between psoriasis and ASCVD risk factors, clinicians should regularly screen psoriasis patients for these risk factors and consistently offer them lifestyle modification advice, including smoking cessation, moderation of alcohol consumption, regular exercise, healthy eating, and weight management. For patients who have coexisting ASCVD risk factor conditions such as hypertension, diabetes mellitus, dyslipidaemia, obesity, chronic kidney disease, and mental health disorders, these diseases should be optimally managed, engaging the relevant specialists when necessary.

Strengths and Limitations

It is important to highlight the strengths of our systematic review and meta-analysis. First, it was a large-scale study of 21 observational studies involving 778,049 patients with psoriasis and 16,881,765 control subjects. The large sample size most likely conferred a high statistical power to our analysis. Second, our review provides the highest level of ‘acceptable’ evidence as regards the association between psoriasis and ASCVD. Although RCTs are higher than observational studies in the hierarchy of evidence, it would be unethical to study the association between psoriasis and ASCVD using an RCT design. Excluding RCTs, the cohort studies provide the highest level of evidence from research. Seventeen out of the 21 studies included in our review were cohort studies. Third, the quality of evidence from our review was high because only studies with a low risk of bias were included. Using the modified Newcastle-Ottawa tool for observational studies [63], the minimum quality score for our 21 included studies was seven.

Despite the strengths of our study, we recognise some limitations, including residual confounding, bias, heterogeneity, and limited data on our ASCVD outcomes. None of the 21 included studies performed a comprehensive covariate adjustment, including controlling for as many ASCVD risk factors as possible. Hence, residual confounding in these studies remains possible and is probably the most important limitation of this review. This potential for residual confounding affects the certainty of the independence of the positive association between psoriasis and ASCVD in our analyses. Furthermore, the various systemic medications used to treat psoriasis subjects in this review may have had a neutral effect or contributed to or offered protection against ASCVD. Since the majority of the included studies did not adjust for anti-psoriasis medication (Table 1), this may have also potentially contributed to residual confounding (positive or negative) in these studies.

The estimates of our pooled analyses may have been affected by bias in the included studies. There was a risk of misclassification bias in these studies. Most relied on the use of diagnostic codes to identify psoriasis groups, control groups, and ASCVD outcomes from medical records and databases. This method may not be free of mistakes such as coding errors and omissions. In addition, accurate diagnoses of psoriasis and ASCVD outcomes require medical practitioners with sufficient experience in the relevant specialities. Inexperienced practitioners may either incorrectly diagnose psoriasis/ASCVD outcomes or miss their diagnoses. Hence, the studies included in this review had risks of misclassifying their study subjects and the ASCVD outcomes. Another potential source of misclassification bias was the method of ascertainment of severe psoriasis in our included studies. Fifteen of our studies had baseline data that classified psoriasis patients according to disease severity, but only a minority of them [53,64] have identified patients with severe disease using formal criteria such as the percentage of BSA affected or the PASI. Most severe psoriasis patients in our review were identified by relying on surrogate measures (such as the use of systemic anti-psoriasis medications, hospitalisation for psoriasis, or the presence of psoriatic arthritis). Noticeably, 13 out of the 15 studies were population-based, and the proportion of psoriasis patients who had severe disease ranged from 3% to 27% (Table 1). However, based on the percentage of BSA affected, an estimated 15% to 20% of patients with psoriasis have moderate to severe disease [89]. Hence, our cohorts who had identified < 10% of their psoriasis groups as having severe disease may have misclassified some severe cases of disease as mild, and this may have ultimately affected the estimates of our pooled analyses in terms of psoriasis severity.

The high heterogeneity observed in most pooled analyses of the cohort studies prevents us from drawing definite conclusions. Although heterogeneity was low-to-moderate in most pooled analyses of the non-cohort studies, we are aware that the Q-statistic and I^2^ index both have low power to detect heterogeneity in a meta-analysis when there are only a few studies [90]. The potential sources of the high heterogeneity in our analyses include the variable settings of the included studies (America, Asia, and Europe), variable study designs (cross-sectional vs case-control; prospective vs retrospective cohort), variable methods of ascertainment of exposure (psoriasis) and ASCVD outcomes among the studies, variable levels of covariate adjustment among the studies, and the variable statistical power of the studies due to sample size variation (Table 1).

Our findings may have also been limited by a lack of data for some of the clinical ASCVD outcomes. None of the studies that met our inclusion criteria had data on atherosclerotic diseases of the extremities, kidneys, or mesentery. Another consideration is that our pooled analyses in terms of the severity of psoriasis have been limited because as many as seven out of our 21 included studies did not have effect sizes for mild and severe psoriasis.

## Conclusions

Within the limits of our analyses, we have demonstrated that psoriasis (both mild and severe) is associated with a significantly increased risk of ASCVD. The management goals for psoriasis in adults should include routine ASCVD risk assessment and prompt implementation of preventive measures for ASCVD. To improve the robustness of evidence from systematic reviews and meta-analyses like ours, future observational studies evaluating the association between psoriasis and ASCVD should strive to limit bias as much as possible and to perform more extensive covariate adjustments.

## Figures and Tables

**Figure 19:**
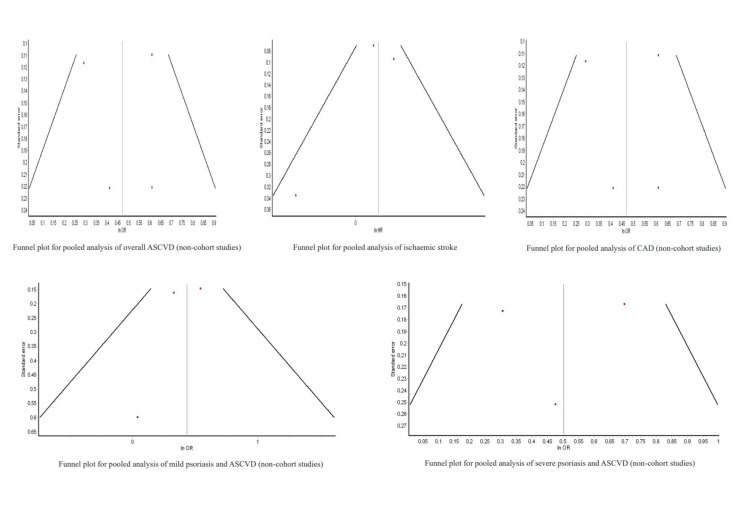
Funnel plots for meta-analyses with less than five studies ASCVD: atherosclerotic cardiovascular disease; CAD: coronary artery disease; OR: odds ratio; HR: hazard ratio

## Appendices

Appendix 1

**Table 5:** Search strategy

Database	Search strategy (medical subject headings (MeSH) terms or medical keywords/synonyms used)
MEDLINE through PubMed	“ischaemic attack, transient” OR “ischaemic stroke” OR “stroke” OR “intracranial arteriosclerosis” OR “carotid stenosis” OR “carotid artery diseases” OR “angina pectoris” OR “angina, stable” OR “angina, unstable” OR “acute coronary syndrome” OR “myocardial infarction” OR “myocardial ischemia” OR “coronary disease” OR “coronary artery disease” OR “aortic aneurysm” OR “mesenteric ischaemia” OR “renal artery obstruction” OR “peripheral arterial disease” OR “arterial occlusive diseases” OR “peripheral vascular diseases” OR “atherosclerosis” OR “cardiovascular diseases” AND ”psoriasis”
EMBASE	“transient ischemic attack” OR “ischemic stroke” OR “cerebrovascular disease” OR “occlusive cerebrovascular disease” OR “cerebrovascular accident” OR “cerebral atherosclerosis” OR “carotid artery disease” OR “carotid atherosclerosis” OR “carotid stenosis” OR “coronary artery disease” OR “coronary atherosclerosis” OR “ischemic heart disease” OR “heart muscle ischemia” OR “heart infarction” OR “acute coronary syndrome” OR “stable angina pectoris” OR “unstable angina pectoris” OR “angina pectoris” OR “aortic aneurysm” OR “aortic atherosclerosis” OR “mesenteric ischaemia” OR “peripheral arterial disease” OR “peripheral arterial occlusion” OR “peripheral vascular disease” OR “artery occlusion” OR “renal artery stenosis” OR “atherosclerotic renal artery stenosis” OR “renal artery occlusion” OR “renal artery disease” OR “atherosclerosis” OR “cardiovascular disease” AND “psoriasis”
Scopus	“transient ischaemic attack” OR “ischaemic stroke” OR “stroke” OR “cerebrovascular disease” OR “cerebrovascular accident” OR “cerebral atherosclerosis” OR “intracranial atherosclerosis” OR “carotid artery disease” OR “carotid disease” OR “carotid atherosclerosis” OR “carotid stenosis” OR “coronary artery disease” OR “coronary heart disease” OR “coronary atherosclerosis” OR “coronary disease” OR “ischaemic heart disease” OR “myocardial ischaemia” OR “myocardial infarction” OR “heart attack” OR “acute coronary syndrome” OR “stable angina” OR “unstable angina” OR “angina” OR “aortic aneurysm” OR “aortic atherosclerosis” OR “mesenteric ischaemia” OR “peripheral artery disease” OR “peripheral arterial disease” OR “peripheral arterial occlusive disease” OR “peripheral vascular disease” OR “arterial occlusive disease” OR “renal artery stenosis” OR “atherosclerotic renal artery stenosis” OR “renal artery obstruction” OR “renal artery disease” OR “atherosclerosis” OR “atherosclerotic” OR “cardiovascular disease” OR “cardiovascular diseases” AND “psoriasis”
BASE	“psoriasis AND transient ischaemic attack”, “psoriasis AND ischaemic stroke”, “psoriasis AND cerebrovascular disease”, “psoriasis AND carotid stenosis”, “psoriasis AND carotid occlusion”, “psoriasis AND carotid artery disease”, “psoriasis AND myocardial infarction”, “psoriasis AND ischaemic heart disease”, “psoriasis AND coronary heart disease”, “psoriasis AND coronary artery disease”, “psoriasis AND acute coronary syndrome”, “psoriasis AND angina”, “psoriasis AND aortic aneurysm”, “psoriasis AND mesenteric ischaemia”, “psoriasis AND renal artery stenosis”, “psoriasis AND peripheral artery disease, “psoriasis AND peripheral arterial disease”, “psoriasis AND peripheral vascular disease”, “psoriasis AND atherosclerotic cardiovascular disease”, “psoriasis AND cardiovascular disease”
Google Scholar	“transient ischaemic attack” OR “ischaemic stroke” OR “stroke” OR “cerebrovascular disease” OR “cerebrovascular accident” OR “cerebral atherosclerosis” OR “intracranial atherosclerosis” OR “carotid artery disease” OR “carotid disease” OR “carotid atherosclerosis” OR “carotid stenosis” OR “coronary artery disease” OR “coronary heart disease” OR “coronary atherosclerosis” OR “coronary disease” OR “ischaemic heart disease” OR “myocardial ischaemia” OR “myocardial infarction” OR “heart attack” OR “acute coronary syndrome” OR “stable angina” OR “unstable angina” OR “angina” OR “aortic aneurysm” OR “aortic atherosclerosis” OR “mesenteric ischaemia” OR “peripheral artery disease” OR “peripheral arterial disease” OR “peripheral arterial occlusive disease” OR “peripheral vascular disease” OR “arterial occlusive disease” OR “renal artery stenosis” OR “atherosclerotic renal artery stenosis” OR “renal artery obstruction” OR “renal artery disease” OR “atherosclerosis” OR “atherosclerotic” OR “cardiovascular disease” OR “cardiovascular diseases” AND “psoriasis”

Appendix 2 

Appendix 3 

**Table 6:** Table of analyses ASCVD, atherosclerotic cardiovascular disease; CI, confidence interval; HCI, higher limit of confidence interval; HR, hazard ratio; LCI, lower limit of confidence interval; N/A, not applicable; OR, odds ratio.

Meta-analysis (and pooled statistics)	Study	HR (or OR)	LCI 95%	HCI 95%	Weight (%)	Sensitivity analysis	Excluded study	Pooled HR (or OR)	LCI 95%	HCI 95%	Cochran’s Q	p	I^2^	I^2^ LCI 95%	I^2^ HCI 95%
Overall ASCVD (cohort studies): HR = 1.21 (95% CI, 1.14 to 1.28); I^2^ = 63.45% (95% CI, 38.48 to 78.28); Cochran's Q = 43.77; Chi^2^, p = 0.00; Tau^2^ = 0.01	Gelfand et al. (2006) [29]	3.12	0.70	13.87	0.16	Overall ASCVD (cohort studies)	Gelfand et al. (2006) [29]	1.21	1.14	1.28	42.23	0.00	64.48	39.46	79.16
	Wakkee et al. (2010) [50]	1.01	0.91	1.12	9.18		Wakkee et al. (2010) [50]	1.23	1.16	1.30	31.51	0.01	52.39	15.83	73.07
	Ahlehoff et al. (2011) [36]	1.34	1.14	1.57	6.64		Ahlehoff et al. (2011) [36]	1.20	1.13	1.28	42.15	0.00	64.41	39.34	79.13
	Lin et al. (2011) [69]	2.10	1.27	3.43	1.30		Lin et al. (2011) [69]	1.20	1.13	1.27	39.02	0.00	61.56	33.81	77.67
	Chiang et al. (2012) [39]	1.27	1.05	1.52	5.72		Chiang et al. (2012) [39]	1.20	1.13	1.28	43.50	0.00	65.52	41.45	79.69
	Dowlatshahi et al. (2013) [53]	0.70	0.46	1.05	1.79		Dowlatshahi et al. (2013) [53]	1.22	1.15	1.29	37.01	0.00	59.47	29.75	76.62
	Levesque et al. (2013) [75]	1.17	1.04	1.31	8.62		Levesque et al. (2013) [75]	1.21	1.14	1.29	43.44	0.00	65.47	41.37	79.67
	Maradit-Kremers et al. (2013) [71]	1.18	0.80	1.74	1.99		Maradit-Kremers et al. (2013) [71]	1.21	1.14	1.29	43.76	0.00	65.72	41.84	79.79
	Dregan et al. (2014) [78]	1.12	0.90	1.38	4.83		Dregan et al. (2014) [78]	1.21	1.14	1.29	43.27	0.00	65.34	41.11	79.60
	Ogdie et al. (2015) [43]	1.09	1.00	1.20	9.81		Ogdie et al. (2015) [43]	1.22	1.15	1.30	38.38	0.00	60.92	32.58	77.35
	Wu et al. (2015) [72]	1.30	1.16	1.47	8.47		Wu et al. (2015) [72]	1.20	1.13	1.28	42.27	0.00	64.51	39.53	79.18
	Chiu et al. (2016) [73]	1.80	1.25	2.61	2.18		Chiu et al. (2016) [73]	1.20	1.13	1.27	39.27	0.00	61.80	34.28	77.79
	Khalid et al. (2016) [68]	1.27	1.11	1.46	7.61		Khalid et al. (2016) [68]	1.20	1.13	1.28	43.26	0.00	65.33	41.09	79.59
	Egeberg et al. (2017) [76]	1.10	0.94	1.29	6.71		Egeberg et al. (2017) [76]	1.22	1.14	1.29	42.36	0.00	64.59	39.68	79.22
	Feldman et al. (2018) [70]	1.23	1.19	1.27	12.25		Feldman et al. (2018) [70]	1.21	1.12	1.30	41.16	0.00	63.56	37.68	78.69
	Leisner et al. (2018) [77]	1.39	1.18	1.65	6.35		Leisner et al. (2018) [77]	1.20	1.13	1.27	41.06	0.00	63.47	37.51	78.64
	Jung et al. (2019) [74]	1.23	1.04	1.45	6.40		Jung et al. (2019) [74]	1.21	1.13	1.29	43.73	0.00	65.70	41.81	79.78
Overall ASCVD (non-cohort studies): OR = 1.60 (95% CI, 1.34 to 1.92); I^2^ = 31.14% (95% CI, 0.00 to 75.21); Cochran's Q = 4.36; Chi^2^, p = 0.23; Tau^2^ = 0.01	Xiao et al. (2009) [67]	1.84	1.48	2.29	36.77	Overall ASCVD (non-cohort studies)	Xiao et al. (2009) [67]	1.45	1.21	1.74	1.65	0.44	0.00	0.00	87.41
	Yang et al. (2011) [35]	1.51	1.02	2.43	14.12		Yang et al. (2011) [35]	1.62	1.29	2.05	4.28	0.12	53.29	0.00	86.61
	Armstrong et al. (2012) [38]	1.84	1.19	2.83	14.17		Armstrong et al. (2012) [38]	1.56	1.25	1.95	3.91	0.14	48.81	0.00	85.10
	Yeung et al. (2013) [64]	1.34	1.07	1.69	34.94		Yeung et al. (2013) [64]	1.78	1.49	2.13	0.66	0.72	0.00	0.00	68.62
Ischaemic stroke: HR = 1.14 (95% CI, 0.96 to 1.36); I^2^ = 43.84% (95% CI, 0.00 to 83.20); Cochran's Q = 3.56; Chi^2^, p = 0.17; Tau^2^ = 0.01	Chiang et al. (2012) [39]	1.27	1.05	1.52	41.41	Ischaemic stroke	Chiang et al. (2012) [39]	0.98	0.64	1.50	2.00	0.16	49.95	0.00	0.00
	Dowlatshahi et al. (2013) [53]	0.69	0.36	1.34	6.38		Dowlatshahi et al. (2013) [53]	1.17	1.04	1.32	1.14	0.29	11.94	0.00	0.00
	Jung et al. (2019) [74]	1.12	0.97	1.28	52.21		Jung et al. (2019) [74]	1.02	0.57	1.81	3.07	0.08	67.40	0.00	92.63
Myocardial infarction (cohort studies): HR = 1.20 (95% CI, 1.10 to 1.31); I^2^ = 60.30% (95% CI, 23.11 to 79.51); Cochran's Q = 25.19; Chi^2^, p = 0.00; Tau^2^ = 0.01	Gelfand et al. (2006) [29]	3.12	0.70	13.87	0.34	Myocardial infarction (cohort studies)	Gelfand et al. (2006) [29]	1.19	1.09	1.30	23.54	0.01	61.77	23.93	80.79
	Wakkee et al. (2010) [50]	0.94	0.80	1.11	11.49		Wakkee et al. (2010) [50]	1.23	1.13	1.33	17.46	0.04	48.45	0.00	75.09
	Ahlehoff et al. (2011) [36]	1.26	1.10	1.45	12.97		Ahlehoff et al. (2011) [36]	1.19	1.08	1.31	24.05	0.00	62.58	25.77	81.14
	Lin et al. (2011) [69]	2.10	1.27	3.43	2.70		Lin et al. (2011) [69]	1.18	1.09	1.28	19.88	0.02	54.73	7.76	77.78
	Levesque et al. (2013) [75]	1.17	1.04	1.31	14.35		Levesque et al. (2013) [75]	1.21	1.09	1.34	25.19	0.00	64.27	29.60	81.86
	Maradit-Kremers et al. (2013) [71]	1.18	0.80	1.74	4.05		Maradit-Kremers et al. (2013) [71]	1.20	1.09	1.31	25.19	0.00	64.27	29.61	81.87
	Ogdie et al. (2015) [43]	1.09	1.00	1.20	15.81		Ogdie et al. (2015) [43]	1.22	1.10	1.35	21.69	0.01	58.51	16.45	79.39
	Wu et al. (2015) [72]	1.30	1.16	1.47	14.16		Wu et al. (2015) [72]	1.18	1.07	1.30	21.81	0.01	58.73	16.96	79.49
	Egeberg et al. (2017) [76]	1.10	0.94	1.29	11.79		Egeberg et al. (2017) [76]	1.21	1.10	1.34	24.48	0.00	63.23	27.24	81.42
	Leisner et al. (2018) [77]	1.39	1.18	1.65	11.27		Leisner et al. (2018) [77]	1.17	1.07	1.28	20.96	0.01	57.06	13.12	78.77
	Jung et al. (2019) [74]	1.45	0.63	3.32	1.06		Jung et al. (2019) [74]	1.19	1.09	1.31	24.94	0.00	63.92	28.81	81.71
Myocardial infarction (non-cohort studies): OR = 1.57 (95% CI, 1.15 to 2.15); I^2^ = 74.15% (95% CI, 0.00 to 94.16); Cochran's Q = 3.87; Chi^2^, p = 0.05; Tau^2^ = 0.04	Xiao et al. (2009) [67]	1.84	1.48	2.29	50.59	Myocardial infarction (non-cohort studies)	N/A	N/A	N/A	N/A	N/A	N/A	N/A	N/A	N/A
	Yeung et al. (2013) [64]	1.34	1.07	1.69	49.41		N/A	N/A	N/A	N/A	N/A	N/A	N/A	N/A	N/A
Coronary artery disease (cohort studies): HR = 1.20 (95% CI, 1.13 to 1.28); I^2^ = 67.03% (95% CI, 42.25 to 81.18); Cochran's Q = 39.43; Chi^2^, p = 0.00; Tau^2^ = 0.01	Gelfand et al. (2006) [29]	3.12	0.70	13.87	0.18	Coronary artery disease (cohort studies)	Gelfand et al. (2006) [29]	1.20	1.13	1.28	37.89	0.00	68.33	43.63	82.20
	Wakkee et al. (2010) [50]	1.01	0.91	1.12	10.57		Wakkee et al. (2010) [50]	1.23	1.16	1.30	26.82	0.01	55.26	16.54	76.02
	Ahlehoff et al. (2011) [36]	1.34	1.14	1.57	7.62		Ahlehoff et al. (2011) [36]	1.19	1.11	1.27	37.88	0.00	68.32	43.62	82.20
	Lin et al. (2011) [69]	2.10	1.27	3.43	1.48		Lin et al. (2011) [69]	1.19	1.12	1.27	34.72	0.00	65.43	37.72	80.81
	Dowlatshahi et al. (2013) [53]	0.70	0.41	1.19	1.30		Dowlatshahi et al. (2013) [53]	1.21	1.14	1.29	35.34	0.00	66.04	38.98	81.11
	Levesque et al. (2013) [75]	1.17	1.04	1.31	9.91		Levesque et al. (2013) [75]	1.21	1.12	1.29	39.05	0.00	69.27	45.54	82.66
	Maradit-Kremers et al. (2013) [71]	1.18	0.80	1.74	2.27		Maradit-Kremers et al. (2013) [71]	1.20	1.13	1.28	39.41	0.00	69.55	46.11	82.80
	Dregan et al. (2014) [78]	1.12	0.90	1.38	5.54		Dregan et al. (2014) [78]	1.21	1.13	1.29	38.90	0.00	69.15	45.30	82.60
	Ogdie et al. (2015) [43]	1.09	1.00	1.20	11.30		Ogdie et al. (2015) [43]	1.22	1.14	1.30	33.78	0.00	64.48	35.76	80.36
	Wu et al. (2015) [72]	1.30	1.16	1.47	9.75		Wu et al. (2015) [72]	1.19	1.11	1.28	38.02	0.00	68.44	43.86	82.26
	Egeberg et al. (2017) [76]	1.10	0.94	1.29	7.71		Egeberg et al. (2017) [76]	1.21	1.13	1.30	37.95	0.00	68.38	43.73	82.23
	Feldman et al. (2018) [70]	1.23	1.19	1.27	14.15		Feldman et al. (2018) [70]	1.20	1.11	1.30	37.46	0.00	67.96	42.89	82.03
	Leisner et al. (2018) [77]	1.39	1.18	1.65	7.28		Leisner et al. (2018) [77]	1.19	1.11	1.27	36.81	0.00	67.40	41.74	81.76
	Jung et al. (2019) [74]	1.33	1.21	1.47	10.94		Jung et al. (2019) [74]	1.19	1.11	1.27	35.68	0.00	66.37	39.64	81.26
Coronary artery disease (non-cohort studies): OR = 1.60 (95% CI, 1.34 to 1.92); I^2^ = 31.14% (95% CI, 0.00 to 75.21); Cochran's Q = 4.36; Chi^2^, p = 0.23; Tau^2^ = 0.01	Xiao et al. (2009) [67]	1.84	1.48	2.29	36.77	Coronary artery disease (non-cohort studies)	Xiao et al. (2009) [67]	1.45	1.21	1.74	1.65	0.44	0.00	0.00	87.41
	Yang et al. (2011) [35]	1.51	1.02	2.43	14.12		Yang et al. (2011) [35]	1.62	1.29	2.05	4.28	0.12	53.29	0.00	86.61
	Armstrong et al. (2012) [38]	1.84	1.19	2.83	14.17		Armstrong et al. (2012) [38]	1.56	1.25	1.95	3.91	0.14	48.81	0.00	85.10
	Yeung et al. (2013) [64]	1.34	1.07	1.69	34.94		Yeung et al. (2013) [64]	1.78	1.49	2.13	0.66	0.72	0.00	0.00	68.62
Aortic aneurysm: HR = 1.45 (95% CI, 1.04 to 2.02); I^2^ = 66.98% (95% CI, 0.00 to 92.53); Cochran's Q = 3.03; Chi^2^, p = 0.08; Tau^2^ = 0.04	Chiu et al. (2016) [73]	1.80	1.25	2.61	37.51	Aortic aneurysm	N/A	N/A	N/A	N/A	N/A	N/A	N/A	N/A	N/A
	Khalid et al. (2016) [68]	1.27	1.11	1.46	62.49		N/A	N/A	N/A	N/A	N/A	N/A	N/A	N/A	N/A
Mild psoriasis and ASCVD (cohort studies): HR = 1.17 (95% CI, 1.08 to 1.26); I^2^ = 74.02% (95% CI, 52.74 to 85.72); Cochran's Q = 38.49; Chi^2^, p = 0.00; Tau^2^ = 0.01	Gelfand et al. (2006) [29]	1.54	1.24	1.91	6.89	Mild psoriasis and ASCVD (cohort studies)	Gelfand et al. (2006) [29]	1.14	1.06	1.23	29.56	0.00	69.56	41.48	84.17
	Ahlehoff et al. (2011) [36]	1.29	1.15	1.45	11.08		Ahlehoff et al. (2011) [36]	1.15	1.06	1.25	31.62	0.00	71.54	45.86	85.04
	Dowlatshahi et al. (2013) [53]	0.77	0.49	1.21	2.47		Dowlatshahi et al. (2013) [53]	1.18	1.09	1.28	35.93	0.00	74.95	53.29	86.57
	Levesque et al. (2013) [75]	1.18	1.05	1.33	10.97		Levesque et al. (2013) [75]	1.17	1.07	1.27	37.45	0.00	75.97	55.48	87.03
	Dregan et al. (2014) [78]	1.03	0.97	1.11	13.22		Dregan et al. (2014) [78]	1.19	1.09	1.30	31.63	0.00	71.54	45.87	85.04
	Ogdie et al. (2015) [43]	1.08	0.98	1.18	12.14		Ogdie et al. (2015) [43]	1.18	1.08	1.30	38.03	0.00	76.34	56.26	87.20
	Wu et al. (2015) [72]	1.31	1.14	1.51	9.93		Wu et al. (2015) [72]	1.15	1.07	1.25	32.97	0.00	72.70	48.41	85.56
	Chiu et al. (2016) [73]	1.73	1.12	2.65	2.67		Chiu et al. (2016) [73]	1.16	1.07	1.25	34.43	0.00	73.86	50.93	86.08
	Khalid et al. (2016) [68]	1.20	1.03	1.39	9.51		Khalid et al. (2016) [68]	1.17	1.07	1.27	37.46	0.00	75.98	55.49	87.03
	Egeberg et al. (2017) [76]	1.03	0.96	1.11	13.02		Egeberg et al. (2017) [76]	1.19	1.09	1.30	32.85	0.00	72.61	48.19	85.51
	Jung et al. (2019) [74]	1.14	0.95	1.37	8.10		Jung et al. (2019) [74]	1.17	1.08	1.28	38.42	0.00	76.58	56.77	87.31
Severe psoriasis and ASCVD (cohort studies): HR = 1.43 (95% CI, 1.23 to 1.65); I^2^ = 65.41% (95% CI, 34.31 to 81.78); Cochran's Q = 28.91; Chi^2^, p = 0.00; Tau^2^ = 0.03	Gelfand et al. (2006) [29]	7.08	3.06	16.36	2.63	Severe psoriasis and ASCVD (cohort studies)	Gelfand et al. (2006) [29]	1.35	1.22	1.50	13.72	0.13	34.39	0.00	68.73
	Ahlehoff et al. (2011) [36]	1.60	1.32	1.96	12.90		Ahlehoff et al. (2011) [36]	1.40	1.19	1.65	25.47	0.00	64.66	30.49	82.03
	Dowlatshahi et al. (2013) [53]	0.55	0.14	2.22	1.07		Dowlatshahi et al. (2013) [53]	1.44	1.24	1.67	27.29	0.00	67.02	35.81	83.06
	Levesque et al. (2013) [75]	1.16	0.94	1.42	12.64		Levesque et al. (2013) [75]	1.47	1.25	1.74	26.59	0.00	66.15	33.85	82.68
	Dregan et al. (2014) [78]	1.29	1.01	1.64	11.56		Dregan et al. (2014) [78]	1.45	1.23	1.72	28.77	0.00	68.72	39.61	83.80
	Ogdie et al. (2015) [43]	1.26	0.92	1.72	9.58		Ogdie et al., (2015) [43]	1.45	1.23	1.71	28.72	0.00	68.67	39.49	83.77
	Wu et al. (2015) [72]	1.28	1.02	1.60	12.08		Wu et al. (2015) [72]	1.46	1.23	1.72	28.69	0.00	68.63	39.41	83.76
	Chiu et al. (2016) [73]	2.15	1.03	4.51	3.24		Chiu et al. (2016) [73]	1.41	1.21	1.63	27.35	0.00	67.09	35.96	83.09
	Khalid et al. (2016) [68]	1.67	1.21	2.32	9.25		Khalid et al. (2016) [68]	1.40	1.20	1.64	27.13	0.00	66.82	35.36	82.97
	Egeberg et al. (2017) [76]	1.21	1.05	1.39	14.57		Egeberg et al. (2017) [76]	1.47	1.24	1.75	25.75	0.00	65.05	31.38	82.20
	Jung et al. (2019) [74]	1.59	1.20	2.10	10.48		Jung et al. (2019) [74]	1.41	1.20	1.66	27.44	0.00	67.20	36.21	83.13
Mild psoriasis and ASCVD (non-cohort studies): OR = 1.54 (95% CI, 1.25 to 1.90); I^2^ = 0.00% (95% CI, 0.00 to 85.00); Cochran's Q = 1.39; Chi^2^, p = 0.50; Tau^2^ = 0.00	Xiao et al. (2009) [67]	1.72	1.29	2.30	53.07	Mild psoriasis and ASCVD (non-cohort studies)	Xiao et al. (2009) [67]	1.36	1.00	1.85	0.22	0.64	0.00	0.00	0.00
	Yang et al. (2011) [35]	1.04	0.32	3.36	3.21		Yang et al. (2011) [35]	1.56	1.26	1.94	0.94	0.33	0.00	0.00	0.00
	Yeung et al. (2013) [64]	1.39	1.01	1.91	43.72		Yeung et al. (2013) [64]	1.67	1.26	2.21	0.66	0.42	0.00	0.00	0.00
Severe psoriasis and ASCVD (non-cohort studies): OR = 1.65 (95% CI, 1.29 to 2.12); I^2^ = 24.72% (95% CI, 0.00 to 92.17); Cochran's Q = 2.66; Chi^2^, p = 0.26; Tau^2^ = 0.01	Xiao et al. (2009) [67]	2.01	1.45	2.79	40.29	Severe psoriasis and ASCVD (non-cohort studies)	Xiao et al. (2009) [67]	1.44	1.09	1.90	0.31	0.58	0.00	0.00	0.00
	Yang et al. (2011) [35]	1.61	0.98	2.63	21.34		Yang et al. (2011) [35]	1.66	1.13	2.43	2.64	0.10	62.16	0.00	91.27
	Yeung et al. (2013) [64]	1.36	0.97	1.91	38.37		Yeung et al. (2013) [64]	1.88	1.43	2.47	0.54	0.46	0.00	0.00	0.00
